# Pregnancy Complicated by Ludwig's Angina Requiring Delivery

**DOI:** 10.1155/2010/158264

**Published:** 2010-06-10

**Authors:** Kathleen Moorhead, Maryam Guiahi

**Affiliations:** ^1^Department of Emergency Medicine, University of Michigan, Ann Arbor, MI 48109, USA; ^2^Division of Family Planning, Department of Obstetrics and Gynecology, Columbia University, New York, NY 10032, USA

## Abstract

At 33 weeks of gestation, a 24-year-old developed Ludwig's angina that worsened despite aggressive therapy. This is the first reported case of Ludwig's Angina in pregnancy that required an emergent cesarean section for fetal indications. Delivery may have contributed to improvement in the mother's health status.

## 1. Introduction

Ludwig's Angina is a rare consequence of periodontal infection that can result in severe upper airway obstruction and potentially death. Prior to our report, there have only been two cases reported in pregnant women. We report the first case that required immediate delivery secondary to worsening fetal and maternal health status.

## 2. Case Report

This patient is a 24-year-old with a history of hepatitis C infection, intravenous drug use, and ten-pack year smoking history who presented as transfer of care during her thirty-third week of gestation. She had two prior deliveries: one vaginal and one cesarean. Initially, she presented with a tooth abscess that was treated with outpatient medication therapy that included oral antibiotic (amoxicillin-clavulanate) and analgesic medications. Despite reported compliance, she represented two days later at a community hospital with a low-grade fever and left-sided neck swelling consistent with angioedema. She was counseled and consented for elective endotracheal intubation given the concern for significant airway compromise. Next, she was given a single dose of dexamethasone to decrease swelling. Her diagnosis was Ludwig's Angina in pregnancy and she was transferred to our tertiary hospital's obstetrical intensive care unit. 

Upon admission, the patient was arousable, responsive to commands, and able to communicate despite the in situ endotracheal tube. She was normotensive, mildly tachycardic, and saturating well on room air without the need for assisted ventilation. Her palate and dentition were not fully visible, but her trachea appeared midline. Her neck exam was firm and tender with massive asymmetric neck swelling. Antenatal testing revealed a reactive fetal heart rate tracing, sonographic fetal biometry was consistent with her dates, and the fetus had a normal anatomic survey. The otolaryngology service at our center confirmed the diagnosis of Ludwig's Angina, initiated parenteral piperacillin-tazobactam therapy, and recommended against steroids to prevent further maternal immunosupression. An incision and drainage of her neck abscess was performed in the operating room followed by removal of her endotracheal tube and placement of a tracheotomy tube. The oral maxillary facial service performed extraction of five of her teeth. A gram stain of the abscess revealed gram-negative rods and her antibiotic therapy was continued. Throughout this therapy, antenatal testing was periodically performed and found to be reassuring. 

On the first postoperative day, secondary to presumed septic shock, she became hypotensive with increasing oxygen demands that required vasopressive and ventilatory support. She was transferred to the medical intensive care unit. Her antibiotic regimen was expanded to include parenteral vancomyocin therapy. She was additionally diagnosed with acute respiratory distress syndrome (ARDS) based on her presentation and chest radiographic findings of bilateral opacities. Despite several efforts, the patient's status did not improve and the fetal heart rate tracing became persistently nonreassuring with repetitive late decelerations. At this time, the maternal fetal medicine specialist proceeded with cesarean delivery for nonreassuring fetal indications. She had an urgent repeat lower transverse cesarean section, delivering a female infant weighing 2120 grams with Apgars of 2, 6, and 7. Neonatal service was in attendance for the delivery. The newborn was intubated, admitted to the neonatal intensive care unit, and started on empiric ampicillin and gentamicin therapy. 

Immediately following delivery of the neonate, the patient's oxygen saturation improved to 98%. The patient returned to the medical intensive care unit. On postpartum day number one, she was weaned off of vasopressor therapy and transitioned to a trach collar. A chest radiograph revealed improved pulmonary status thirty-two hours after delivery. The tracheotomy was first used continuously for five days, then intermittently, and successfully removed on postpartum day number eight. The newborn did well in the neonatal intensive care unit: her blood cultures were negative, antibiotics were discontinued, she was extubated on day of life 2, and she was progressively weaned off of oxygen therapy. The patient was then discharged home on postoperative day nine with oral antibiotic therapy and plans for follow-up. Her daughter was subsequently discharged home on day of life 15. Both mother and baby had no other complications.

## 3. Discussion

First recognized in 1836 by Dr. Karl Ludwig who observed deadly forms of tooth infection, Ludwig's angina is a rare case found in medicine [1]. Typically, as an odontogenic infection of the 2nd and 3rd molars, it has direct spread, rather than hematogenous or lymphatic, to the submandibular, sublingual, and submental spaces. This can lead to rapid and profound neck swelling and in some cases death by airway compression. The infection and edema are limited by the deep cervical fascia, mandible, and hyoid. As a result, the tongue and floor of the mouth are elevated and posteriorly displaced with compromise of the airway, which can result in abrupt asphyxiation. Infection often results from streptococci, staphylococci, or bacteroides, although 50% of infections are polymicrobial. Management of a patient with Ludwig's angina requires early aggressive therapy with antibiotics, incision and drainage of any abscess, and protection of the airway [1, 2].

Fifty percent of pregnant women annually in the United States are affected by periodontal disease involving chronic anaerobic oral infection [3]. The pregnant patient with a maxillofacial infection requires special attention given the physiologic changes of pregnancy, which puts the patients at increased risk of complications. Such changes include increased sodium retention, dilutional anemia, decreased colloid pressure, and an upper airway that is affected by capillary engorgement and subsequent edema of the nasal passages, larynx, oropharynx, and trachea. Chronic gingivitis, friable oral mucosa, and mild immunosuppresion of pregnancy play an important role in outcome. Further, changes in gastrointestinal motility and other physiologic systems put the pregnant patient at increased risk of acute respiratory distress syndrome, pneumonia, sepsis, amniotic fluid embolism, disseminated intravascular coagulation, and pulmonary edema secondary to aspiration. Adverse pregnancy outcomes such as preterm birth, low birth weight, fetal growth restriction, preeclampsia, and perinatal mortality are associated with periodontal disease [3–6].

There are only 2 prior case reports of pregnant women with Ludwig's Angina in the present literature, neither of which required delivery as part of the treatment plan [7, 8]. In our case, delivery was indicated given the nonreassuring fetal tracing that was likely associated with fetal hypoxia or acidosis. The mother's health status immediately improved with delivery. The physiological changes associated with this preterm delivery may have resulted in improved ventilation status. Functional residual capacity (FRC) decreases on average by 18% in the third trimester and is further compromised in the case of ARDS [9]. Given the increase in FRC that occurs postpartum, this improvement in gas exchange may have improved her hypoxemic state [9]. Respiratory compliance may also improve post delivery following a downward displacement of the diaphragm [10]. A case series of 10 intubated pregnant patients demonstrated a 28% reduction in fraction of inspired oxygen requirement by 24 hours after delivery [11]. Delivery is also associated with improved hemodynamic changes, specifically related to cardiac output, when the delivery of the neonate relieves compression placed on the vena cava [12]. Broad-spectrum antibiotic coverage, including the addition of vancomycin therapy, likely contributed to resolution of this patient's septic condition. Adequate serum levels of antibiotic therapy may have coincided with delivery.

It is unclear as to whether these physiological changes imply a direct cause and effect relationship for the improvement of our patient's health status. It is controversial whether pregnant patients with acute respiratory failure such as ARDS will improve with delivery in situations when immediate delivery is not indicated [10, 11]. Delivery should continue to be reserved for obstetrical indications [10, 11]. For this rare antepartum medical presentation of Ludwig's angina, the decision to perform a cesarean section avoided a potentially fatal outcome for the neonate. Delivery may have contributed to improvement in the mother's health status.
